# Ultramicronized Palmitoylethanolamide (um-PEA): A New Possible Adjuvant Treatment in COVID-19 patients

**DOI:** 10.3390/ph14040336

**Published:** 2021-04-06

**Authors:** Annalisa Noce, Maria Albanese, Giulia Marrone, Manuela Di Lauro, Anna Pietroboni Zaitseva, Daniela Palazzetti, Cristina Guerriero, Agostino Paolino, Giuseppa Pizzenti, Francesca Di Daniele, Annalisa Romani, Cartesio D’Agostini, Andrea Magrini, Nicola Biagio Mercuri, Nicola Di Daniele

**Affiliations:** 1 UOC of Internal Medicine-Center of Hypertension and Nephrology Unit, Department of Systems Medicine, University of Rome Tor Vergata, 00133 Rome, Italy; giul.marr@gmail.com (G.M.); dilauromanuela@gmail.com (M.D.L.); annapietroboni@icloud.com (A.P.Z.); daniela.palazzetti96@gmail.com (D.P.); cristinaguerriero@hotmail.it (C.G.); francesca.didaniele@gmail.com (F.D.D.); didaniele@med.uniroma2.it (N.D.D.); 2 Neurology Unit, Department of Systems Medicine, University of Rome Tor Vergata, 00133 Rome, Italy; maria.albanese@hotmail.it (M.A.); mercurin@med.uniroma2.it (N.B.M.); 3 PhD School of Applied Medical, Surgical Sciences, University of Rome Tor Vergata, 00133 Rome, Italy; 4 Department of Biomedicine and Prevention, University of Rome Tor Vergata, 00133 Rome, Italy; agostino.paolino.pv@gmail.com (A.P.); andrea.magrini@uniroma2.it (A.M.); 5 School of Specialization in Geriatrics, University of Rome Tor Vergata, 00133 Rome, Italy; giusypizzenti@gmail.com; 6 PHYTOLAB (Pharmaceutical, Cosmetic, Food Supplement, Technology and Analysis), DiSIA, University of Florence, Sesto Fiorentino, 50019 Florence, Italy; annalisa.romani@unifi.it; 7 Department of Experimental Medicine, University of Rome Tor Vergata, 00133 Rome, Italy; cartesio.dagostini@ptvonline.it; 8 Laboratory of Clinical Microbiology, Policlinico Tor Vergata, 00133 Rome, Italy; 9 IRCCS Santa Lucia Foundation, 00179 Rome, Italy

**Keywords:** COVID-19, SARS-CoV-2, ultramicronized palmitoylethanolamide, organ damage, neuroinflammation, adjuvant treatment

## Abstract

The Coronavirus Disease-19 (COVID-19) pandemic has caused more than 100,000,000 cases of coronavirus infection in the world in just a year, of which there were 2 million deaths. Its clinical picture is characterized by pulmonary involvement that culminates, in the most severe cases, in acute respiratory distress syndrome (ARDS). However, COVID-19 affects other organs and systems, including cardiovascular, urinary, gastrointestinal, and nervous systems. Currently, unique-drug therapy is not supported by international guidelines. In this context, it is important to resort to adjuvant therapies in combination with traditional pharmacological treatments. Among natural bioactive compounds, palmitoylethanolamide (PEA) seems to have potentially beneficial effects. In fact, the Food and Drug Administration (FDA) authorized an ongoing clinical trial with ultramicronized (um)-PEA as an add-on therapy in the treatment of Severe Acute Respiratory Syndrome Coronavirus-2 (SARS-CoV-2) infection. In support of this hypothesis, in vitro and in vivo studies have highlighted the immunomodulatory, anti-inflammatory, neuroprotective and pain-relieving effects of PEA, especially in its um form. The purpose of this review is to highlight the potential use of um-PEA as an adjuvant treatment in SARS-CoV-2 infection.

## 1. Introduction

In late 2019, an unknown pneumonia started to spread, with initial cases found in the town of Wuhan, China. It was later identified as the cause in a new beta-coronavirus, a member of the Coronaviridae family, called Severe Acute Respiratory Syndrome Coronavirus-2 (SARS-CoV-2), which has shown important similarities with two other members of that family, SARS-CoV (which caused an outbreak in 2002–2003) and the Middle East Respiratory Syndrome Coronavirus Infection (MERS-CoV; found in an outbreak from the Middle East in 2012) [1,2]. SARS-Cov-2 has rapidly spread from China around the world, thanks to human-to-human transmission and its high contagiousness, causing a disease called Coronavirus Disease-19 (COVID-19), currently responsible for over 100 million infections and 2 million deaths worldwide in more than 200 countries [3].

There is enormous variability regarding the clinical manifestations of COVID-19. In fact, affected subjects can be asymptomatic or show symptoms that are mild, moderate, or severe (which can have bad outcomes). The most common symptoms are represented by fever, dry cough, muscle aches, “shortness of breath”, and headache, while symptoms such as rhinorrhea, sore throat, and those affecting the gastrointestinal system are rarer [4]. More severe complications include respiratory failure, neurological symptoms, acute kidney injury (AKI), shock, and death [5].

The lungs are the most affected organs. Respiratory symptoms are certainly the most frequent; however, other organs can be affected, and there are numerous extrarespiratory manifestations [6]. Cardiac complications such as acute heart failure, myocarditis, arrhythmia, and shock [7], gastrointestinal symptoms such as diarrhea, nausea, vomiting, and abdominal pain [8], liver damage with high levels of aspartate aminotransferase (AST) and alanine aminotransferase (ALT) [9], and cutaneous alterations such as rash and skin lesions [10] have been reported in COVID-19 patients. Furthermore, neurological manifestations like hyposmia, hypogeusia, headache, and dizziness have also been documented [11]. Meanwhile, acute cerebrovascular diseases, impaired consciousness, and epilepsy have been occasionally described in COVID-19 patients [12]. All the severe organ damages observed in SARS-CoV-2 infection are mainly characterized by an inflammatory process [13]. The kidney is another organ particularly involved in the course of SARS-CoV-2 infection; in fact, numerous cases of AKI have been recorded, which increase the risk of death by 3 times compared to COVID-19 patients without renal involvement [14].

Currently, there are no totally effective antiviral drugs against SARS-CoV-2. The drugs currently in use are designed to counteract the symptoms of infection and are based on anti-inflammatory and immunomodulatory effects and on antiviral actions observed in in-vitro studies. These drugs are not specifically for the treatment of COVID-19 and have numerous limitations in their use [15]. For this reason, in addition to traditional drugs, a possible adjuvant treatment could be represented by natural bioactive compounds (NBCs). Nowadays, there are numerous studies investigating the possible role of oral food supplements based on NBCs in alleviating COVID-19 symptoms. Among these, a very interesting one is palmitoylethanolamide (PEA), an amide of palmitic acid with well-known anti-inflammatory properties, on which this review focuses.

## 2. Ultramicronized-PEA and its Mechanisms of Action

**Figure 1:**
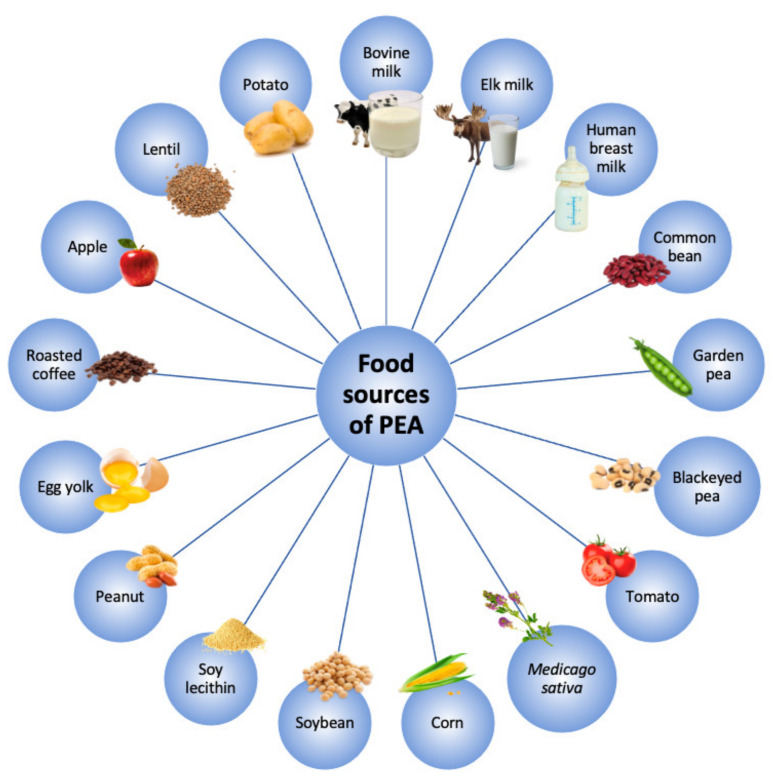
Main food sources of palmitoylethanolamide (PEA).

PEA is an amide of endogenous fatty acid of the *N*-acylethanolamine family, naturally produced in the body and largely found in several food sources, as reported in Figure 1 [16]. The food content of PEA varies from 950.000 ng·g^−1^ in soy lecithin to 0.25 ng·g^−1^ in bovine milk [17].

PEA is a cannabimimetic compound that performs a wide variety of biological functions to counteract chronic pain and inflammation [18,19,20,21,22,23]. In the current state of knowledge, PEA is considered by the international scientific community as an oral food supplement with immunomodulatory, anti-inflammatory, neuroprotective, and pain-relieving properties [22]. Historically, between the 1960s and 1970s, PEA was used in the drug “Impulsin” to treat flu and cold, proving to be especially effective in prophylaxis and in the treatment of upper respiratory tract infections [24,25].

Nowadays, in the literature, the available data on PEA pharmacokinetics and bioavailability are few [17]. An interesting animal-model study on PEA pharmacokinetics showed that after oral administration, the drug reaches its highest concentration in 15 min; 2 h later, it comes back to basal values [26]. Subsequently, to enhance the biodisponibility of PEA, two new formulations were synthesized: micronized-PEA and ultramicronized-PEA (um-PEA). In particular, um-PEA displays better bioavailability compared to its native form, with improved effectiveness [27]. In fact, in an animal-model, um-PEA concentration was higher, up to six times, after 1–2 h from oral administration compared to the native form [28].

The main um-PEA immunomodulatory effect seems to be related to mast cells [29,30], which are typically involved in respiratory infections caused by coronaviruses and/or other influenza viruses [31,32]. This mechanism of action was first hypothesized by Rita Levi Montalcini et al., who described the downregulation of mast cells via the “autacoid local injury antagonism” [33].

um-PEA is able to inhibit the release and action of mast cells, reduce the expression of cyclooxygenase-2 (COX-2) and inducible nitric oxide synthase (iNOS) [34], and counteract the release of proinflammatory proteases, histamine, cytokines, and chemokines (also implicated in COVID-19) [35,36]. Moreover, um-PEA exerts its immunomodulatory action as an agonist, binding to peroxisome proliferator-activated receptor alpha (PPARα), which is expressed at the level of different organs but especially in the immune cells such as monocytes, macrophages, and T- and B-lymphocytes [37]. In fact, as already underlined, um-PEA regulates the hyperactivation of mast cells [38] by reducing their migration and degranulation, and it mediates the overactivation of both astrocytes and glial cells [29,30]. These actions are responsible for numerous pathological conditions such as those related to asthma and allergies [39], fibromyalgia [40], and chronic lung diseases [41]. For these reasons, um-PEA may exert respiratory protective effects, making it a promising candidate for the adjuvant treatment of COVID-19 [25].

PEA is also able to interact with cannabinoid receptors [42,43], ATP-sensitive K+ and transient receptor potential vanilloid type-1 (TRPV1) channels [44,45], nuclear factor kappa-light-chain-enhancer of activated B-cells (NF-kB) [46], G-protein-coupled receptors 55 (GPR55) [47], and PPARα [48], exerting anti-inflammatory and pain-relieving actions [19,49]. In particular, um-PEA, having a chemical structure similar to classic endocannabinoids, enhances their effects, counteracting their catabolism and increasing their concentration (*entourage effect*) [42,50]. This PEA-specific action is mainly mediated by the TRPV1 channel, whose activation seems to be indirectly induced by two different mechanisms. The first one is related to the *entourage effect* [51]; meanwhile, the second is mediated by PPARα [52].

In addition, um-PEA is able to decrease hyperalgesia as it can reduce COX-2 and iNOS gene transcription and restore the action of PPARα at the dorsal root ganglia level. In fact, um-PEA seems to inhibit the degradation of IkB-α and the nuclear translocation of p65 NF-κB, acting as a transcription modulator for the attenuation of peripheral hyperalgesia, with inhibition of the release of inflammatory cytokines such as tumor necrosis factor-α (TNF-α) and some interleukins (ILs) [25,46,53]. Further action of um-PEA can be related to direct activation at low doses of the orphan GPR55 receptor, largely expressed in many brain areas and in the gastrointestinal system [17,54,55]. Although GPR55 physiological action is not completely known, it seems to act through the activation of several mediators (such as Gq G12, RhoA, actin, PLC, and IP(3)R-gated stores), enhancing intracellular calcium [56].

New evidence shows that in the case of COVID-19, mast cells are activated by Toll-like receptors (TLRs), increasing inflammation in the lungs and inducing fibrosis [34,57]. um-PEA treatment may be able to modulate the mechanisms linked to PPARα and TLRs that are involved in SARS-CoV-2 infection [58]. In addition to the actions described above, um-PEA is also able to reduce oxidative stress and improve endothelial damage, the intestinal barrier integrity, and brain function [59,60,61,62,63].

In this regard, the consequent positive role of um-PEA on the central nervous system (CNS) is represented by the reduction of neuroinflammation, acting on the mast cell–microglia axis [42,50]. Through the activation of PPARα, um-PEA is able to stimulate the synthesis of neurosteroids [48]. This action would seem to be regulated by two mechanisms. The first is characterized by molecular control through high conductance potassium channels (IKCa and BKCa), which, once opened, trigger the silencing of neuronal activation. The second one consists of activating gene transcription, with subsequent neurosteroid synthesis [64,65].

In recent years, several studies have investigated how the um-PEA compound may exert a neuroprotective action [17,66]. In vitro and in vivo studies have shown um-PEA’s ability to stimulate neurogenesis and promote the release of neurotrophic factors [67]. In fact, um-PEA is able to interact with some GPRs to stimulate the ligand-activated nuclear receptor PPARα subtype, which promotes the regulation of lipids, carbohydrates, and amino acid metabolism and of inflammation mediators [68]. PPARα is widely expressed at the glial level, where it plays a protective role in the suppression of neuroinflammation and the inhibition of oxidative stress [69,70].

**Figure 2:**
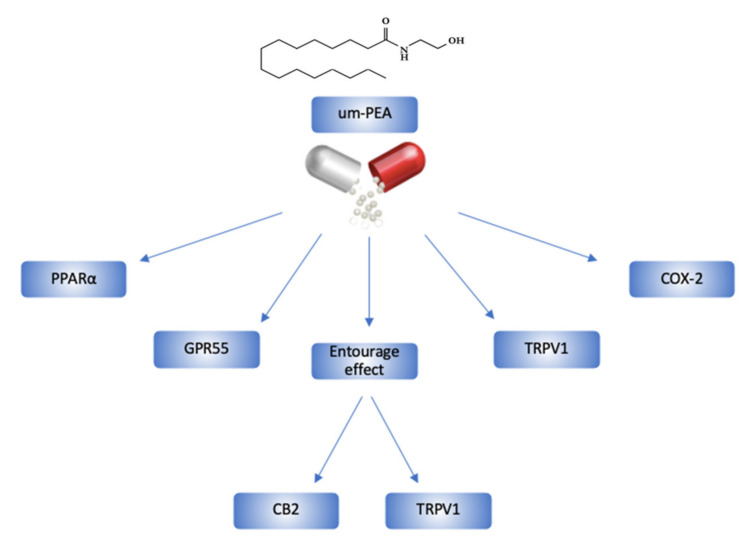
um-PEA’s chemical structure and its mechanism of action in the human body. CB2, cannabinoid 2; COX-2, cyclooxygenase-2; GPR55, G-protein-coupled receptors 55; PPARα, peroxisome proliferator-activated receptor α; TRPV1, transient receptor potential vanilloid type-1.

Activation of PPARα, present on astrocytes, regulates the activity of modulating genes for both the expression of glial glutamate transporters and the transcription of neurosteroids (first of all, allopregnanolone) [65,71] (Figure 2).

## 3. Pathogenic Mechanisms of SARS-CoV-2

SARS-CoV-2 is a single-stranded RNA β-coronavirus formed by an envelope containing a lipid membrane and protein components (the spike (S) glycoprotein, the envelope (E) protein, and the membrane (M) protein). The genomic component of single-stranded RNA is bound to the nucleocapsid (N) protein. The S protein needs to be cleaved into S1 and S2 portions by transmembrane serine protease 2 (TMPRSS2) to be functional and to penetrate host cells. The S1 portion binds the receptor angiotensin-converting enzyme 2 (ACE2) that induces receptor-mediated endocytosis of the virion into the host cell. The binding affinity between S-protein and ACE-receptors is 10–20-fold higher compared to other coronaviruses; this affinity explains the easier human-to-human transmission [72,73].

The most important way of spreading SARS-CoV-2 is through droplets and direct contact with mucosa, but many studies have pointed out other transmission ways, such as fomites, aerosol, and transplacental via birth [74].

Although in the beginning, SARS-CoV-2 was considered a respiratory disease, it has been demonstrated that the virus can infect many kinds of human cells because of the presence of a great number of ACE-receptors in many organs. In nasal mucosa, high levels of angiotensin-converting enzyme 2 (ACE2) expression were found in secretory goblet cells, and this may explain the high tropism of SARS-CoV-2 for this type of cells [75]. The virus starts its replication in the upper respiratory tract, and in up to 20% of patients, the infection spreads to the lungs, where the epithelial cells represent the most important target due to the high expression of ACE2 and TMPRSS2 receptors. Moreover, it has been demonstrated that the virus also infects macrophages. This leads to the apoptosis of lung epithelial cells, which triggers an important inflammatory response, with the recruitment of immune cells that eliminate the infected cells [76]. This phase represents the start of the “cytokine storm”, which has been pointed out in the most-ill COVID-19 patients. The spreading to the immune cells during the inflammatory response represents the main cause of alveolar damage and hemorrhage. Moreover, interstitial modifications have been reported in in-vitro studies [77].

Although the COVID-19 initial phase occurs in the lungs, studies have shown that the virus can spread to many other organs and tissues [78]. An important contribution to COVID-19 immunopathology is given by the spreading of the virus into vessels and blood cells. It can easily enter the platelets because of the high expression of ACE2 and TMPRSS2 receptors on the surface of these cells [79,80]. Moreover, the interaction between viral S-protein and the platelets’ glycoproteins can directly induce platelet activation that results in the release of coagulation factors and cytokines; this, in turn, contributes to thrombosis and hemostasis. The latter are caused by the adhesion of activated platelets to the subendothelium. In the lung, the hyperactivity of the platelets contributes to the enhancement of the inflammatory response, inducing vessel ischemia and embolism through the activation of the coagulation cascade [81]. Currently, it has been confirmed that the most important cause of death due to SARS-CoV-2 infection is related to coagulopathy induced by the inflammatory response, which ends with disseminated intravascular coagulation (DIC) [82].

In addition to alveolar damage that can potentially become critical, above all, in elderly and immunosuppressed patients and in the presence of comorbidities (such as cancer, obesity, metabolic syndrome, cardiovascular diseases), SARS-CoV-2 infection can involve many other tissues and organs since this virus has a wide organotropism.

It has been pointed out that through the olfactory nerve starting from the nasal epithelium, the virus can invade the CNS, which can also be reached hematogenously [83]. Moreover, ACE2 has also been found at high rates in different neurological cells, such as neurons and glial cells. Nonetheless, ACE2 and TMPRSS2 receptors can be found on the surface of cardiomyocytes, kidney cells, the liver, and the pancreas, in the gastrointestinal tract, and at very high rates in the gallbladder [84].

Recent studies have focused on how SARS-CoV-2 systemic infection causes endothelial dysfunction and coagulopathy. In this context, systemic inflammation and cytokine release seem to play important roles in the onset of pneumonia and other symptoms. The importance of the “cytokine storm” has been pointed out in many studies [13,85,86]: once activated, the “endothelium dysfunction–platelets activation–inflammatory response–rise of cytokines releasing-tissue injury” loop can easily spread to many organs and tissues.

## 4. Main Organ Damage Induced by SARS-CoV-2

**Figure 3:**
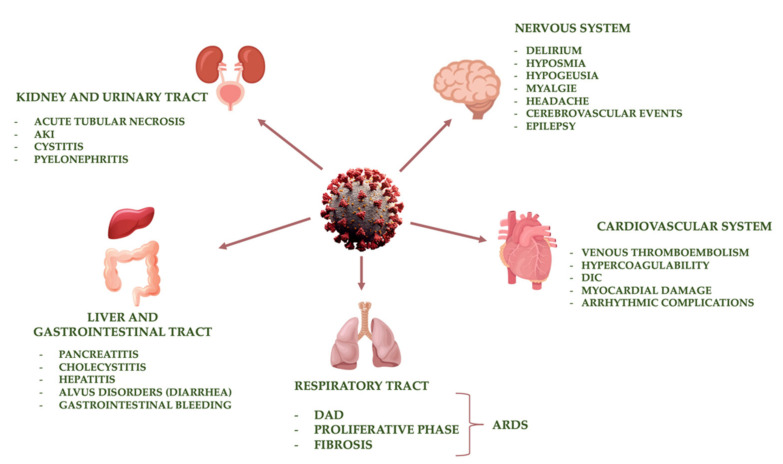
Main target organs of SARS-CoV-2 infection. Abbreviations: AKI, acute kidney injury; ARDS, acute respiratory distress syndrome; DAD, diffuse alveolar damage; DIC, disseminated intravascular coagulation.

In the early stages, SARS-CoV-2 infection manifests itself clinically with muscle aches, headache, fatigue, diarrhea, chills, fever, and cough. Thereafter, the patients may present respiratory symptoms such as “shortness of breath” and dry cough. Some subjects may get worse with severe pneumonia. Pneumonia can subsequently progress to acute respiratory distress syndrome (ARDS) and multiorgan failure (MOF), leading to patient death [87]. The severity of the disease depends on several factors, such as age and the presence of other comorbidities. The mechanism of SARS-CoV-2 infection is determined by binding with ACE2, through which the virus enters host cells. ACE2 is present in the respiratory, intestinal, renal, and cardiovascular systems and the immune cells. Consequently, associated with SARS-CoV-2, there may be a number of complications that do not exclusively affect the respiratory system but also the cardiovascular, gastrointestinal, and urinary systems (Figure 3) [88].

### 4.1. Respiratory Tract

One of the fastest and most dangerous manifestations of SARS-CoV-2 infection is pulmonary damage, probably because of the high presence of ACE2 receptors on the alveolar surface of type II pneumocytes [89,90]. In the initial phase of infection, the virus leads to lower-airway damage [91]. Most patients with pulmonary involvement will soon show diffuse alveolar damage (DAD) [92], including alveolar edema and hemorrhage, fibrin exudation in alveolar spaces, bronchiolar damage, and hyaline membrane formation that is linked to epithelial cell necrosis [93]. According to the severity of COVID-19 disease, a second phase, characterized by a proliferative path, can follow the first—it includes tissue alterations such as the formation of fibromyxoid-organizing exudates, hyperplasia (especially of type II pneumocytes), and the widening of septae [94]. The inflammatory and proliferative response can subsequently induce a third fibrotic phase that can be pointed out in most patients who have died from severe pneumonia and ARDS induced by SARS-CoV-2 [95].

The initial apoptosis and the necrosis of pulmonary cells trigger an inflammatory response that increases tissue damage. This leads to the activation of the “cytokines storm”, characterized by the acute release of proinflammatory cytokines, such as IL-6, IL-1, TNF-α, and interferon, that are produced by macrophages, mast cells, and endothelial cells [96]. Released cytokines recall macrophages, neutrophils, and T-lymphocytes to the infection site, inducing and amplifying tissue damage [78,97]. During COVID-19, the inflammatory process alters the antithrombotic endothelial protective mechanisms. These include the release of nitric oxide with vasodilatory action, the secretion of prostaglandin (PG)I2, which inhibits the recruitment and activation of leukocytes, and, finally, the presence on the endothelial surface of CD39 receptors, which inhibit platelet aggregation. Therefore, the endothelial damage induced by SARS-CoV-2 leads to the loss of such protective mechanisms, provoking thromboembolic phenomena [98]. Thromboembolism plays a key role in inducing lung damage and injury at other tissue levels: in the early stage, the coagulation is activated by the immunological response and leads to the thrombosis of both small arteries and veins, causing successive pulmonary embolism [99]. The absence of blood flow contributes to tissue damage and necrosis, and represents one of the first triggers to the worsening of clinical features, characterized by the reduction of oxygen saturation due to, in turn, a significant ventilation and perfusion mismatch. Moreover, in alveolar sacs, an environment able to foster bacterial and fungal superinfections can be established, as demonstrated in several autopsies [93]. Secondly, bronchopneumonia is often caused by *Pseudomonas aeruginosa*, sp., *Aspergillus* sp., and cytomegalovirus [93,100].

Lung injury can sometimes be diagnosed and followed-up using X-ray and CT imaging, even if several studies have suggested that radiological features can underline histological alterations [101,102]. In addition, SARS-CoV-2 lung injury does not always show typical and defined pictures. However, the imaging role in the diagnosis and assessment of virus-induced injury is still very important [103]. Lung consolidation and ground-glass opacities at X-ray/CT imaging represent the most common path of COVID-19 infection [104], sometimes accompanied by reticular opacities. Moreover, multifocal airspace can be pointed out in COVID-19 pneumonia, frequently in a bilateral and basal distribution. Peripheral lung engagement is one of the most specific “pieces” of radiological evidence of SARS-CoV-2 infection, although other inflammatory processes can lead to multifocal or confluent injuries [104].

### 4.2. Cardiovascular System

SARS-CoV-2, in addition to respiratory complications, induces alterations in the cardiovascular system. In fact, numerous studies have shown a link between COVID-19 and cardiovascular complications such as arterial hypertension, heart failure, myocardial infarction, myocarditis, arrhythmias, coagulopathy, venous thromboembolism, and DIC [105]. Clinically, cardiovascular symptoms can present as an initial manifestation or appear later. In a study by Kui et al., it was observed that 7% of COVID-19 subjects experienced heart palpitations as an initial symptom [106]. In another study, 16% of the patients underwent different degrees of myocardial damage [107].

In this context, subjects with previous cardiovascular diseases have an increased risk of severity and mortality in the case of SARS-CoV-2 infection due to the massive presence of ACE2 receptors in the cardiovascular system [108]. Indeed, the measurement of plasma angiotensin peptides and plasma ACE2 levels may be useful in evaluating treatment efficacy and the status of the renin–angiotensin–aldosterone system in COVID-19 patients [109].

The etiology of ACE2-dependent cardiovascular damage induced by COVID-19 appears to be complex. Metabolic alterations, hypoxia, and inflammation of the myocardium play an important role in the pathophysiology of myocardial damage and arrhythmic complications. The virus enters the cardiovascular cells through ACE2 receptors. In fact, the increased expression of ACE2 receptors in the cardiovascular system is one of the determinants of infection, causing systemic inflammation and damage to heart tissue. In a postmortem study conducted by Lindner et al. on 39 patients who died from SARS-CoV-2 infection, it was demonstrated that the virus is not localized exclusively in cardiomyocytes but also in interstitial cells or macrophages that invade myocardial tissue [110]. Several epidemiological studies have demonstrated that viral RNA infection induced cardiomyocyte apoptosis, activating the innate immune response, which, through the production of inflammatory cytokines, destabilized the coronary plaques and caused left ventricular insufficiency [111,112]. Furthermore, the role of hyperactivated T-lymphocytes in cardiomyocytic damage has been demonstrated. In fact, there is an immunological dysregulation characterized by inflammation and a “cytokine storm” that contributes to damage on the cardiovascular level [113].

Patients with cardiovascular complications showed significant increases in cardiac troponin I, N-terminal pro-B-type natriuretic peptide, IL-6 and other cytokines (like IL-1B, IL-1RA, IL-7, IL-8, IL-9, IL-10, and TNF-α), C-X-C motif ligand 10 (CXCL10), chemokine ligand 2 (CCL2), and granulocyte-macrophage colony-stimulating factor (GM-CSF) in the bloodstream [114,115].

Coagulopathy associated with SARS-CoV-2 is revealed mainly as organ damage, while hemorrhages are rare. In addition, it is characterized by an increase in D-dimer and the degradation products of fibrin/fibrinogen. Alterations in hemostatic biomarkers indicate that COVID-19 is characterized by massive fibrin formation. This suggests that hyperfibrinolysis secondary to the coagulation process is the primary manifestation of COVID-19-associated coagulopathy [116].

Instead, the prolongation of prothrombin time (PT) and partial thromboplastin time and the reduction of antithrombin activity induce, less frequently, thrombocytopenia in COVID-19 patients compared to other septic DIC patients. In a meta-analysis conducted by Lippi et al. [117], thrombocytopenia has been shown to be associated with an increased risk of disease severity and mortality in COVID-19. Furthermore, thrombocytosis has been found in moderately severe cases and long-term hospitalized patients [118]. Therefore, it might be that thrombocytosis and thrombocytopenia are associated with different stages or severity of the disease.

The mechanisms underlying coagulopathy are not yet fully understood. It is hypothesized that the main causes of coagulation abnormalities are the excessive production of proinflammatory cytokines, increased levels of damage-associated molecular patterns (DAMP), stimulation of cell death mechanisms, and vascular endothelial damage [1]. The increase and dysregulation of inflammatory cytokines and chemokines induce the recruitment of immune cells in infected tissues, which have a defensive role in the host but can also cause damage.

Wu et al. [119] analyzed the relationship between coagulopathy and the development of ARDS in COVID-19 patients, demonstrating that coagulopathy occurs mainly in critically ill patients. Therefore, continuous monitoring of D-dimer and PT is crucial for patient management.

Indeed, the incidence of thrombosis and thromboembolic consequences is more frequent in intensive care COVID-19 patients rather than in non-COVID-19 septic patients in intensive care [120].

### 4.3. Kidney and Urinary Tract

Among the SARS-CoV-2 target organs, the urinary system may also be involved. In particular, in the most critically ill COVID-19 patients, cases of AKI have been reported, with an incidence ranging from 0.9% to 29% of total cases [121]. In detail, the incidence of AKI is more than 20% in hospitalized patients and more than 50% in intensive care patients [122,123]. In addition, an interesting study reported that 60% of hospitalized patients showed proteinuria and 48% showed hematuria [124].

From autopsy reports performed in the city of Wuhan, microscopic examination of the kidney revealed that AKI was induced by acute tubular necrosis, characterized by lumen dilation, vascular degeneration, and alteration of the tubular epithelium [121,125].

It has been hypothesized that the physiopathological mechanisms underlying the onset of AKI are multifactorial [122] and divisible into direct and indirect ones. Recent studies have shown that the virus can directly induce histopathological alterations in the kidney. This hypothesis was supported by a postmortem study showing the presence of viral particles at the level of the tubular epithelium and podocytes, detected by electron microscopy [126,127]. Several studies have demonstrated that the virus enters the renal parenchyma through binding to ACE2, TMPRSS2, and catepsine-1 (CTSI) receptors that are highly expressed at the kidney level. An additional risk factor is the induction of endothelial dysfunction by SARS-CoV-2, interconnected with coagulopathy, typical of COVID-19 patients. Both of the alterations mentioned above are related to the direct viral activation of the complement. In addition, kidney involvement can also be explained by the acute inflammatory state related to SARS-CoV-2 infection, defined as a “cytokine storm”. The increase in cytokines is not constant in all SARS-CoV-2 patients; in fact, it has been hypothesized that they have moderately increased cytokines compared to what is observed in other coronavirus-induced respiratory infections (such as SARS and MERS) [128,129,130].

Indirect mechanisms of renal involvement are partly correlated with clinical manifestations due to infection, i.e., they arise if patients have gastrointestinal symptoms (such as diarrhea and, more rarely, vomiting) and fever, which induce a reduction of volemia, with greater susceptibility to developing prerenal-AKI. At the same time, the drugs used for critically ill patients can be nephrotoxic; antibiotics, especially, can induce tubular damage or acute interstitial nephritis [131,132,133]. Sometimes the most critical COVID-19 patients may have concomitant secondary infections (e.g., bacterial or fungal) that increase the risk of secondary AKI to the septic state. Finally, it should not be neglected that patient age and basal comorbidities play a key role in the onset of AKI [134].

It should also be considered that COVID-19 patients are more susceptible to acute pyelonephritis [135]. Specifically, this association is not supported by consistent epidemiological studies. A combination of SARS-CoV-2 infection and urological symptoms, not necessarily induced by SARS-CoV-2, has been observed. This association turned out to be speculative [136]. In patients with COVID-19, initial symptoms of urological relevance such as flank pain, urinary urgency, pollakiuria, stranguria, and fever have been described [137]. Moreover, it has been hypothesized that these symptoms are attributable to viral cystitis from SARS-CoV-2, although it is not clear if the virus replicates directly in endothelial cells or if it induces endothelitis in the urinary system [138]. The debate remains open.

### 4.4. Liver and Gastrointestinal Tract

ACE2 is also highly expressed in the small and large intestines. In fact, SARS-CoV-2 was found in the cytoplasm of the epithelia of the duodenum and rectum [139], while the expression of ACE2 receptors is significantly lower in the esophagus and stomach [72,140]. Furthermore, in esophageal and gastric mucosa, there is a lower expression of TMPRSS2 compared to intestinal mucosa [141]. From a diagnostic point of view, stool examination showed the presence of SARS-CoV-2 using the reverse transcriptase–polymerase chain reaction (RT-PCR) technique [142].

Clinically, COVID-19 patients have also shown gastrointestinal symptoms such as vomiting, abdominal pain, and diarrhea, as highlighted by several studies [142,143,144]. In fact, in a study conducted by Han et al. [145], it was observed that 19% of patients had diarrhea as the first symptom of the disease, and 62% had fever and enteric symptoms. Furthermore, the authors found that patients with digestive symptoms took a longer period of time between the symptom onset and viral clearance.

During the COVID-19 pandemic, gastrointestinal emergencies have been rare. Some patients have experienced severe diarrhea, with electrolyte disturbances or bloody and inflammatory diarrhea, during or before the onset of pulmonary symptoms [146,147].

In addition, cases of acute pancreatitis and acute cholecystitis induced by SARS-CoV-2 have been reported [148,149,150]. The involvement of the gallbladder and biliary tract in SARS-CoV-2 infection can be explained by the presence of ACE2 receptors on the epithelial cells of the gallbladder and bile ducts.

Gastrointestinal bleeding due to COVID-19 is not very common [151]. In fact, in a study conducted by Yang et al., only 4% of the patients experienced gastrointestinal bleeding [152]. Often, the cause of the bleeding is not identified because endoscopic diagnostic procedures were not performed and patients were treated conservatively [153]. In a study conducted by Massironi et al., endoscopy showed herpetic-like erosions of gastrointestinal mucosa and ulcers with positive biopsies for SARS-CoV-2 [154]. In the case of lower gastrointestinal bleeding, it is probably caused by ischemia related to thrombotic dysfunction, resulting from systemic inflammation or a state of hypoperfusion [155]. The increased levels of D-dimer and fibrinogen present in COVID-19 patients may be responsible not only for pulmonary and peripheral thrombotic phenomena but also intestinal hypercoagulability leading to ischemic events [156,157].

Liver damage is usually associated with low albumin values and increased ALT, AST, and bilirubin levels; meanwhile, high elevated gamma-glutamyl transferase (GGT) levels are observed only in severe cases [144]. On the other hand, alkaline-phosphating (AKP) levels remain unchanged regardless of infection severity [158].

Furthermore, the degree of liver damage seems to be related to disease severity [158]. ACE2 receptors are widely distributed in liver tissue, particularly in bile duct cells and hepatocytes. The biliary duct cells have a high number of ACE2 receptors on their surface, suggesting that hepatocytes are not directly involved in liver damage [158]. The presence of viral load in liver cells was confirmed using the RT-PCR technique, but it was lower compared with biliary duct cells [158]. The imaging and histopathological alterations of COVID-19 patients also include low lobular and portal activity and microvascular steatosis.

Further studies would need to show whether liver damage is solely due to virus entry or whether there is a hepatotoxic effect linked to antiviral drugs.

### 4.5. Nervous System

**Figure 4:**
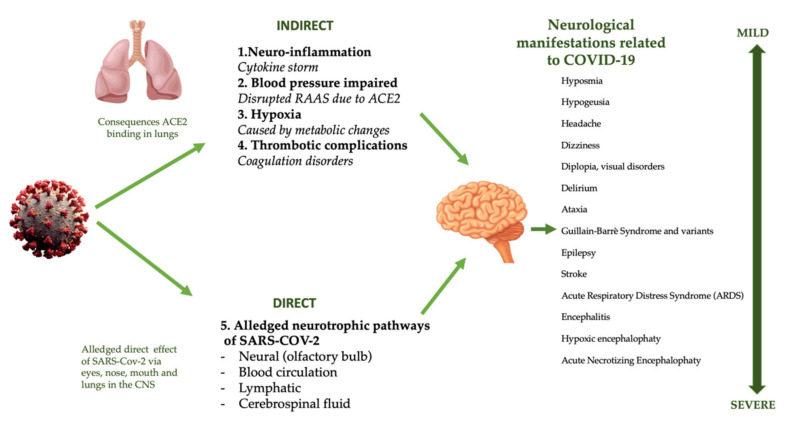
Direct and indirect mechanisms of COVID-19 CNS damage. Abbreviations: ACE2, angiotensin-converting enzyme 2; CNS, central nervous system; RAAS, renin-angiotensin aldosterone system; SARS-CoV-2, severe acute respiratory syndrome coronavirus 2.

Numerous studies have shown that beta-coronaviruses, in particular SARS-CoV, MERS-Cov, and the coronavirus responsible for porcine hemagglutinating encephalomyelitis (HEV), are able to invade the CNS (Figure 4) [159,160,161,162].

The exact mechanism by which SARS-CoV-2 affects the nervous system is not yet fully understood [163]. Overall, the expression of ACE2 in the nervous system is low, so low colonization by SARS-CoV-2 in the CNS and the liver should be assumed [164]. In the CNS, ACE2 receptors are expressed mostly on neurons and glial cells, through which the virus is able to penetrate those cells [83]. The virus colonizes the CNS through different ACE2-binding pathways. The first one is represented by the binding of SARS-CoV-2 with ACE2 receptors on the capillary endothelium, which causes vascular dysfunction, rupture of the blood–brain barrier, and brain invasion [165]. The second mechanism is represented by a direct hematogenous route at the level of circumventricular pathways, such as the subfornical organ, the paraventricular nucleus, the nucleus of the solitary tract, and in the ventrorostral medullary area. These areas are not “protected” by the blood–brain barrier and are rich in ACE2 [166], favorable conditions for the entry into the CNS of any neurotrophic virus [167].

A further mechanism is represented by via transsynaptic signaling, as reported by Lì et al. The authors conducted a study on rats, in which low concentrations of SARS-CoV-2, deposited in the nasal mucosa, accessed the brain through cranial nerves— mainly the olfactory one, invading the CNS and, in particular, the thalamus and brainstem [159,168,169]. These data highlight the marked neurotropism of SARS-CoV-2, subsequently confirmed through (i) autopsy examinations that detected tissue edema, neuronal necrosis, neuroglia alterations; (ii) radiological analysis showing signs of necrotizing encephalitis at the thalamic level and of the brainstem; (iii) fluid cerebrospinal analysis (CSF), with positive genotyping of SARS-CoV-2 in cases of encephalitis [170,171,172].

An additional alternative mechanism of virus penetration into brain tissue is represented by trigeminal and vagal pathways [173,174,175]. The presence of the virus in the brainstem involves viral penetration into the nucleus of the solitary tract, in the baroreceptor and chemoreceptor centers implied in the regulation of respiration and cardiocirculatory functions. A possible retrograde invasion from the brainstem to the lung has also been speculated, indicating a vicious circle that is able to support and aggravate respiratory insufficiency [159].

Clinically, neurological manifestations may precede the appearance of classic respiratory symptoms by a few days [176]; these can be grouped into three categories: (i) central nervous system symptoms (such as dizziness, headache, vascular–cerebral disease, seizures, altered consciousness), (ii) peripheral nervous system symptoms (such as hyposmia, hypogeusia, visual impairment, neuropathic pain, Guillain-Barre syndrome and its variants), (iii) skeletal–muscle injury symptoms (such as myalgia, fatigue, increased levels of muscle enzymes) [176].

Up to date, the most frequent neurological symptoms reported by COVID-19 patients are hyposmia and hypogeusia, continuous headaches, a generalized confusional state, and psychotic disorders.

Other neurological characteristic symptoms are delirium and cognitive deficits. The latter are caused by systemic inflammation associated with prolonged hypoxia, which induces uncontrolled neuroinflammation, representing a pivotal factor of hippocampus and cortical area (centers of cognitive function) damage [177].

The “cytokines storm” characteristic of COVID-19 patients [1] induces immune dysregulation and neurotoxicity [176]. This systemic inflammatory cascade is characterized by a massive release of interleukins and chemokines, which causes a significant rupture of the blood–brain barrier and is responsible for subsequent neuroinflammatory processes [178,179]. Furthermore, the breaking of the blood–brain barrier aggravates neuroinflammation through the activation of TLRs on the microglia by ILs, which then recruit astrocytes, monocytes, dendritic cells, and lymphocytes that have already invaded the CNS [180]. All these cells enhance the neuroinflammatory process, as evidenced by glial cell hypertrophy and by neuronal element death [181,182]. In physiological conditions, the glial cells constantly monitor the cerebral microenvironment and, through the release of cytotoxic substances such as reactive oxygen species, proteinases, and inflammatory mediators, try to limit the spread of infection [183]. This hypothesis of “uncontrolled neuroinflammation” is supported by the fact that the average age of COVID-19 symptomatic patients is over 60 years old [74]. Several studies have reported that patients aged over 60 could already present low-grade brain aging, so that the SARS-CoV-2 infection may worsen the pre-existing clinical picture [181,184,185].

The hypoxia induced by COVID-19 stimulates several transcription factors—first of all, Nf-kβ, and then, hypoxia-inducible factor (HIF). They, in turn, activate genes that regulate the transcription of numerous inflammatory messengers, causing further gliosis [186]. An additional worsening of neuroinflammatory processes is certainly determined by the physical and psychological stress induced by the pandemic [187].

Usually, antiviral immunity can effectively antagonize viral diffusion through the activation of glial cells and the entry of sentinel T-cells into the brain [188]. Unfortunately, in COVID-19 disease, the marked lymphopenia and the possible dysfunction of glial cells, which, in turn, are infected, can cause reduced activation of the defense mechanism, resulting in longer agent persistence in the CNS [13].

Moreover, COVID-19 can cause an increase of other inflammatory markers, such as D-dimer and calcitonin gene-related peptide (CGRP), which play key roles in the pathogenesis of cerebrovascular events and migraine, respectively [189,190,191,192,193]. The last mechanism may be represented by cerebral hypoxia/anoxia, neuronal edema secondary to DAD, and interstitial lung involvement [165]. It is also important to highlight that a large number of patients who experienced both neuroinflammatory symptoms and ARDS (not particularly severe) can continue to present, over time, cognitive impairments [194].

## 5. Conclusions

um-PEA would seem to have numerous healthy effects on different organs and systems, thanks to its various mechanisms acting on inflammation, pain and improving a wide variety of signs and symptoms of both chronic and acute pathological conditions, among them COVID-19 [53]. In this perspective, as um-PEA is safe, endogenous, and nontoxic, its supplementation, aiming at the modulation of the immune system, could represent an add-on therapy to traditional pharmacological drugs for COVID-19 patients [20,22]. Currently, Food and Drug Administration Agency (FDA) has given permission the ongoing phase 2 clinical trial to assess um-PEA effects on hospitalized COVID-19 patients [59,195]. In Italy, a clinical study is in progress (approved by the Ethical Committee of Policlinico Tor Vergata Hospital, Rome, protocol number R.S. 73.20) to evaluate the possible beneficial physiological effects of um-PEA on inflammatory indices in asymptomatic and paucisymptomatic COVID-19 patients.

## Abbreviations List

**ACE2**	Angiotensin-Converting Enzyme
**AKI**	Acute Kidney Injury
**AKP**	Alkaline Phosphating
**ALT**	Alanine Aminotransferase
**ARDS**	Acute Respiratory Distress Syndrome
**AST**	Aspartate Aminotransferase
**CCL2**	Chemokine Ligand 2
**CGRP**	Calcitonin Gene-Related Peptide
**CNS**	Central Nervous System
**COVID-19**	Coronavirus Disease-19
**COX-2**	Cyclooxygenase-2
**CSF**	Fluid Cerebrospinal Analysis
**CTSI**	Catepsine-1
**CXCL10**	C-X-C motif ligand (CXCL)10
**DAD**	Diffuse Alveolar Damage
**DIC**	Disseminated Intravascular Coagulation
**FDA**	Food and Drug Administration Agency
**GGT**	Gamma-Glutamyl Transferase
**GM-CSF**	Granulocyte-Macrophage Colony Stimulating Factor
**GPR55**	G-Protein-Coupled Receptors 55
**HEV**	Porcine Hemagglutinating Encephalomyelitis
**HIF**	Hypoxia-Inducible Factor
**IKCa**	High Conductance Potassium Channels
**IL**	Interleukin
**iNOS**	Inducible Nitric Oxide Synthase
**MERS-CoV**	Middle East Respiratory Syndrome Coronavirus Infection
**MOF**	Multiorgan Failure
**NBC**	Natural Bioactive Compound
**Nf-kβ**	Nuclear Factor Kappa-Light-Chain-Enhancer of Activated B Cells
**PEA**	Palmitoylethanolamide
**PG**	Prostaglandin
**PPARα**	Peroxisome Proliferator-Activated Receptor α
**PT**	Prothrombin Time
**RT-PCR**	Reverse Transcriptase Polymerase Chain Reaction
**SARS-CoV-2**	Severe Acute Respiratory Syndrome Coronavirus-2
**TLRs**	Toll-Like Receptors
**TMPRSS2**	Transmembrane Serine Protease 2
**TNF-α**	Tumor Necrosis Factor-Α
**TRP**	Transient Receptor Potential
**um-PEA**	Ultramicronized Palmitoylethanolamide
